# Effect of the caries-protective self-assembling peptide P11-4 on shear bond strength of metal brackets

**DOI:** 10.1007/s00056-020-00247-1

**Published:** 2020-09-02

**Authors:** Thomas Knaup, Heike Korbmacher-Steiner, Anahita Jablonski-Momeni

**Affiliations:** Dental School, Dept. of Orthodontics, Philipps-University Marburg, Georg-Voigt Str. 3, 35039 Marburg, Germany

**Keywords:** Oral hygiene, Orthodontic appliances, fixed, Dental white spots, Dental enamel, Dental caries, prevention, Mundhygiene, Festsitzende kieferorthopädische Apparaturen, Dentale White Spots, Zahnschmelz, Kariesprävention

## Abstract

**Purpose:**

During orthodontic treatment with fixed appliances, demineralization around brackets often occurs. The aim of this in vitro study was to investigate the effect of the caries-protective self-assembling peptide P11‑4 (SAP P11-4) on the shear bond strength of metal brackets.

**Methods:**

In all, 45 extracted human wisdom teeth were available for the study. The teeth were randomly divided into 3 groups (each *n* = 15) and pretreated as follows: test group 1: application of SAP P11‑4 (Curodont Repair, Windisch, Switzerland) and storage for 24 h in artificial saliva; test group 2: application of SAP P11‑4; control group: no pretreatment with SAP P11‑4. A conventional metal maxillary incisor bracket (Discovery, Dentaurum, Ispringen) was adhesively bonded to each buccal surface. The shear bond strength was tested according to DIN 13990. After shearing, the Adhesive Remnant Index (ARI) was determined microscopically (10 × magnification). Analysis of variance (ANOVA) was used to check the groups for significant differences (α = 0.05). The distribution of the ARI scores was determined with the χ ^2^ test.

**Results:**

There was no significant difference in shear forces between the groups (*p* = 0.121): test group 1 = 17.0 ± 4.51 MPa, test group 2 = 14.01 ± 2.51 MPa, control group 15.54 ± 4.34 MPa. The distribution of the ARI scores between the groups did not vary (*p*-values = 0.052–0.819).

**Conclusion:**

The application of the caries protective SAP P11‑4 before bonding of brackets did not affect the shear bond strength. Therefore, pretreatment of the enamel surface with SAP P11‑4 shortly before bracket insertion can be considered.

## Abstract (de)

**Ziel:**

Während einer kieferorthopädischen Behandlung mit festsitzenden Apparaturen können in der Bracketzirkumferenz Demineralisationen entstehen. Im Rahmen dieser In-vitro-Studie sollte der Einfluss des kariesprotektiven Peptids P11‑4 auf das Haftverhalten zwischen Metallbrackets und Zahnoberfläche untersucht werden.

**Methoden:**

Fünfundvierzig extrahierte humane Weisheitszähne standen für die Studie zur Verfügung. Die Zähne wurden randomisiert auf 3 Gruppen verteilt (je *n* = 15) und unterschiedlich vorbehandelt: Testgruppe 1: Applikation von P11‑4 (Curodont Repair, Windisch, Schweiz) und Lagerung für 24 h in künstlichem Speichel; Testgruppe 2: Applikation von P11‑4; C = Kontrollgruppe: keine Vorbehandlung. Auf jede Bukkalfläche wurde ein konventionelles, metallisches Twinbracket (Discovery, Dentaurum, Ispringen) adhäsiv befestigt. Die Scherhaftfestigkeit wurde nach DIN 13990 mit einer Universalprüfmaschine untersucht. Nach dem Abscheren wurde der ARI (Adhesive Remnant Index) mikroskopisch bestimmt (Vergr. 10:1). Mittels ANOVA (Analysis of Variance) wurden die Gruppen auf signifikante Unterschiede überprüft (α = 0,05). Die Verteilung der ARI-Scores wurde mit dem χ^2^-Test ermittelt.

**Ergebnisse:**

Es bestand kein statistisch signifikanter Unterschied in Bezug auf den Haftverbund zwischen den Untersuchungsgruppen (*p* = 0,121): Testgruppe 1 = 17,0 MPa (Standardabweichung [SD] 4,51), Testgruppe 2 = 14,01 MPa (SD 2,51), Kontrollgruppe 15,54 MPa (SD 4,34). Die Verteilung der ARI-Scores zwischen den Gruppen war statistisch nicht signifikant unterschiedlich (*p*-Werte 0,052–0,819).

**Schlussfolgerung:**

Die Anwendung des kariesprotektiven Peptids P11‑4 vor der regulären Schmelzätztechnik zeigte weder sofort nach der Applikation noch nach 24 h Lagerung in künstlichem Speichel einen signifikanten Einfluss auf den Haftverbund von Brackets. Somit kann eine Vorbehandlung der Schmelzoberfläche mit P11‑4 unmittelbar vor Bracketinsertion in Betracht gezogen werden.

## Introduction

Dental caries is a biofilm-mediated, multifactorial, noncommunicable disease resulting in net mineral loss of dental hard tissues and is determined by biological, behavioral, psychosocial, and environmental factors [30, 42]. Orthodontic treatments, especially with fixed appliances, provide a hurdle to oral hygiene and lead to patients becoming high-risk caries patients. During an orthodontic treatment, plaque that accumulates around the brackets is not removed to the same level as would be required, resulting in demineralization. Consequently initial lesions form around the brackets [38].

The demineralization of the enamel leads to an altered light refraction, which causes a whitish, opaque surface and can have an impact on the esthetic outcome of the orthodontic treatment. It is reported that white spot lesions have a limited ability to improve after appliance removal and can be detectable many years after treatment [46]. The incidence of new carious lesions that formed during orthodontic treatment was found to be 45.8% [47] and development of cavitations after treatment with multibracket appliances was reported in 26.9% of patients [17]. It is even stated that the high treatment demand and occurrence of biofilm-related complications make orthodontic treatment a potential public health threat [44].

Therefore, the emphasis in the prevention of caries during orthodontic treatment should be on management strategies which focus on the remineralization of the lesions, e.g., the use of topical fluoride [13, 20, 21] and amorphous calcium phosphate [35]. Moreover, oral hygiene instructions and regular professional cleanings with motivation of patients have been recommended to inhibit demineralization [32]. The use of reminder systems to improve oral hygiene and adherence to appointments can reduce prevalence of white spot lesions [33].

The use of enamel sealant is another common approach for the prevention of initial lesions. These are used to apply a protective layer to the smooth surfaces and fissures. Various mechanisms of action are known: remineralization of tooth enamel [28], prevention of the formation of a biofilm [11, 22], and the formation of a barrier between enamel and the dental plaque [49]. The sealants may or may not contain fluorides [50] and are used before or after bracket application [4, 7, 8, 18, 19, 37]. The use of such sealants appears to have a caries-preventive effect although the effectiveness seems to vary depending on the sealant used [12, 39, 48, 51].

Despite the efforts described in the literature, the formation of white spot lesions remains a clinical issue within orthodontic treatment and new ways should be sought to prevent caries. One such novel approach is the self-assembling peptide (SAP) technology, which was recently shown to promote the regeneration of enamel within the depth of the carious lesion [2, 10, 23, 25, 26] and inhibit demineralization in high caries risk clinical situations [24]. From the data recently published on SAP P11‑4, it can be concluded that the application of SAP P11‑4 makes the enamel surface more resistant towards caries and acid attack in general. Previous studies have shown SAP P11‑4 to have a positive influence on the bonding of composite resins onto carious dentine if used in combination with an etch-and-rinse system [5, 6].

The present study aimed to investigate the influence of SAP P11‑4 on shear bond strength of metal brackets to the enamel surface. The hypothesis was that there is no significant influence of SAP P11‑4 on shear bond strength.

## Materials and methods

### Sample preparation

In all, 45 extracted human unerupted third molars were included in this in vitro study. The use of extracted teeth was approved by the Ethics Committee of the medical faculty of the Philipps-University Marburg (Ref. No. 107/12). Before the surgical removal of the teeth, each patient was informed and consent was obtained for the use of the teeth for study purposes. The surface of each tooth was examined under a stereomicroscope (Leica MS 5, Leitz, Wetzlar, Germany) at 16 × magnification and samples with mineralization disorders or damage caused by the extraction procedure were not included. The teeth were stored in a 0.5% chloramine T solution directly after extraction and were cleaned after one week.

### Sample treatment and bracket placement

The samples were stored in deionized water according to DIN ISO 3696 at 4 °C. Teeth were embedded in a polytetrafluoroethylene (PTFE) ring using a colorless cold-curing plastic (Technovit 4004, Heraeus, Hanau, Germany), whereby the buccal enamel surface was aligned parallel to the planned shear direction. Teeth were randomly assigned to three groups (*n* = 15 in each group). In test group 1, SAP P11‑4 (Curodont Repair, Windisch, Switzerland) was applied after cleaning and enamel conditioning according to the manufacturer’s instruction. In detail, the enamel was wiped with sodium hypochlorite (2%), and etched with 36% phosphoric acid (Gel etch, Ormco Pty. Ltd., Orange, CA, USA) for 20 s. The enamel was rinsed and SAP P11‑4 was applied via the supplied applicator sponge. SAP P11‑4 was left for 5 min and the samples where then stored for 24 h in artificial saliva. After renewed enamel conditioning with 36% phosphoric acid, a conventional metal maxillary incisor bracket (Discovery, Dentaurum, Ispringen, Germany) was adhesively attached to each buccal surface (Transbond™XT, 40 s light curing with Elipar™, both materials 3M Unitek, Seefeld, Germany). In test group 2, SAP P11-4 was applied as described for group 1 SAP P11-4 then a metal bracket was applied after 5 min, followed by renewed enamel conditioning. In the control group, brackets were adhesively fixed after conditioning as previously described. All samples were stored in ultrapure water of quality 3 according to DIN ISO 3696 (37 ± 2 °C) for 24 h.

### Debonding

**Fig. 1:**
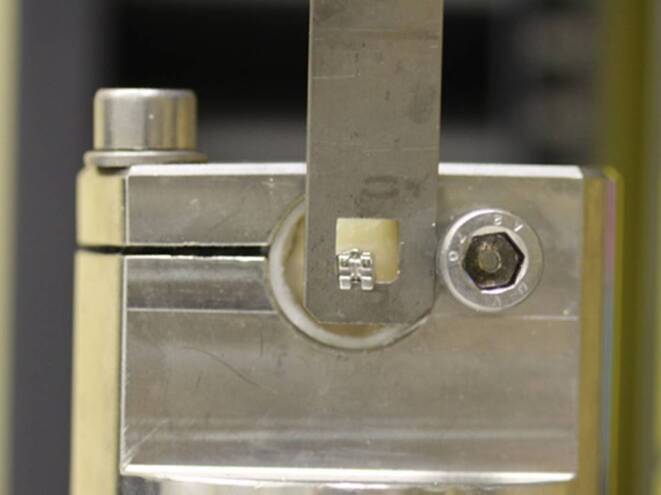
Processing of a specimen. The load of the brackets was conducted with the help of the Zwick universal testing machine in occlusal–gingival direction Testung einer Probe. Die Belastung des Brackets erfolgte durch die Universalprüfmaschine Zwick in okklusal-gingivaler Richtung

All specimens were tested using a standardized and computer-controlled hydraulic testing machine (Zwick 1120.5®, Zwick, Germany; Fig. 1) according to DIN 13990. Each specimen was positioned so that the bonding surface between bracket and enamel was aligned parallel to the occlusal-to-gingival shear force. Shear forces were measured by the force sensor of the testing machine during each test (traverse speed 1 mm/min) until the compound broke and were continuously recorded in a force–displacement diagram. To convert the forces into MPa values, the adhesive surface of the brackets of 13.12 mm (according to manufacturer’s specifications) was used.

### Adhesive remnant index

After removal of the brackets, the remaining residual adhesive material on each tooth surface was determined according to the Adhesive Remnant Index (ARI) [3, 34] by two examiners under 10 × magnification in a light microscope (Leica Z6 APO, Leica Microsystems GmbH, Wetzlar Germany). The ARI was categorized as follows: score 0 = no adhesive on the enamel surface; grade 1 and 2 = less or more than 50% of the residual adhesive on the enamel surface; grade 3 = entire adhesive on the enamel surface. If ARI assessment differed between assessors, a consensus decision was made.

### Statistical evaluation

The statistical evaluation was performed using MedCalc statistical software (v 17.4). Data were tested for normal distribution using the Shapiro–Wilk’s test. The values were normally distributed (*p* > 0.05) and analysis of variance (ANOVA) was used to analyze the groups for significant differences. The distribution of the ARI scores was determined with the χ^2^ test. The significance level was set at α = 0.05 for all tests.

According to the DIN 13990 standard (DIN: Deutsche Institut für Normung), a minimum of 10 samples in each group was required to be included in the study. Preliminary unpublished data showed no significant differences between groups (*n* = 13 in each group) with a pooled standard deviation of 4.35 MPa (95% confidence interval [CI] of difference −4.53; 2.51). Based on these calculations a number of 15 samples were included in each group to increase the power.

## Results

**Fig. 2:**
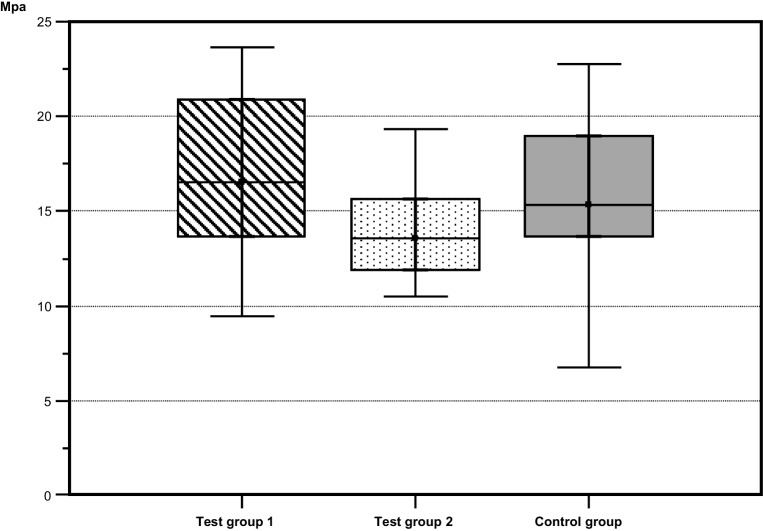
Boxplots of the shear bond strength (MPa) measured for the specimens in all groups. Test group 1: application of P11‑4, 24 h storage in artificial saliva, bracket insertion; test group 2: application of P11‑4, bracket insertion; control group: no pretreatment with P11‑4 prior to bracket insertion Boxplots der Scherhaftfestigkeit (MPa) für die Proben in allen Gruppen. Testgruppe 1: Auftragen von P11‑4 und Lagerung für 24 h in künstlichem Speichel + Bracketapplikation;  Testgruppe 2: Auftragen von P11-4 + Bracketapplikation; Kontrollgruppe: keine Vorbehandlung mit P11‑4 vor Bracketapplikation

During the debonding tests, no bracket or enamel fractures were observed. Mean values for the sheer bond strength were as follows: test group 1: 17.00 ± 4.51 MPa, test group 2: 14.01 ± 2.51, and control group: 15.54 ± 4.34. Test group 1 and 2 did not show significant differences from the control group (*p* = 0.121). The original hypothesis is not rejected. The corresponding boxplots are presented in Fig. 2.

**Fig. 3:**
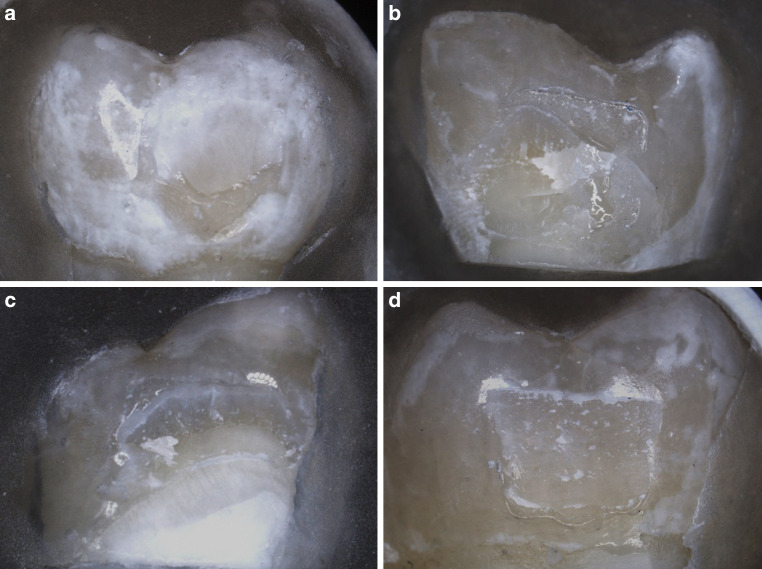
Representative surface images for each Adhesive Remnant Index (ARI) score: **a** ARI score 0, **b** ARI score 1, **c** ARI score 2, **d** ARI score 3 Repräsentative Aufnahmen für jeden ARI(Adhesive Remnant Index)-Score: **a** ARI-Score 0, **b** ARI-Score 1, **c** ARI-Score 2, **d** ARI-Score 3

Adhesive Remnant Index (ARI) values are displayed in Table 1. For all groups the ARI scores were predominantly 1 and 2, with only one specimen in test group 2 having a score of 0 and all groups having a small number of ARI 3. Test group 1 and 2 and control group did not show significant differences between the distribution of the ARI scores: test group 1 vs. test group 2: *p* = 0.143, test group 1 vs. control group: *p* = 0.052 and test group 2 vs. control group: *p* = 0.819. Representative surface images for each ARI score are presented in Fig. 3.

**Table 1:** Distribution of the Adhesive Remnant Index (ARI) scores in each group with 10 × magnification Verteilung der ARI(Adhesive Remnant Index)-Scores in jeder Gruppe nach Betrachtung mit einer Vergrößerung von 10:1

ARI score	Test group 1: P11‑4, 24 h storage in artificial saliva, bracket insertion	Test group 2: P11‑4, bracket insertion	Control group: no pretreatment with P11‑4 prior to bracket insertion
	*N* (%)	*N* (%)	*N* (%)
0: no adhesive on the enamel surface	0 (0)	1 (6.7)	0 (0)
1: less than 50% of the residual adhesive on the enamel surface	6 (40)	6 (40)	6 (40)
2: more than 50% of the residual adhesive on the enamel surface	8 (53.3)	6 (40)	6 (40)
3: entire adhesive on the enamel surface	1 (6.7)	2 (13.3)	3 (20)
*N* (%) total	15 (100)	15 (100)	15 (100)

## Discussion

The present study was the first to evaluate the influence of the self-assembling peptide SAP P11‑4 on the shear bond strength of metal brackets. The study was conducted in accordance with the DIN standard 13990 for better comparison with other studies. Two test groups were included to evaluate whether there was an effect in the shear bond strength when the SAP P11‑4 was applied directly before etching and insertion of the bracket or whether a remineralization period of 24 h would be rational prior to bracket bonding. No significant influence was identified on the bonding of a metal bracket to the enamel surface treated with SAP P11‑4 either directly before or with a 24 h remineralization period in between.

For a clinically sufficient bond between tooth and metal bracket, values between 5.9 and 7.9 MPa were reported by Reynolds [45] and between 5 and 10 MPa by Diedrich [15]. The adhesive values found in our study were between 14.01 and 17 MPa. Hence, clinical sufficiency is therefore given. Previous studies showed that other sealants also had no negative effect on the adhesive bond strength [9, 16, 29, 31, 40]. A further clinical examination of the effectiveness of SAP11‑4 as a bracket environment sealing is still pending.

Many studies focused on the influence of different pretreatment procedures on the shear bond strength such as sandblasting [14, 43] or application of different remineralization agents [36]. The sandblasting of enamel prior to bracket placement is commonly used to increase the shear bond strength and as a measure to reduce bracket failure rate. However, there are heterogeneous results about its effectiveness. In a study by Daratsianos et al. [14] sandblasting could not substitute acid etching and did not offer improved shear bond strength when used before acid etching. Reicheneder et al*. *[43] found increased shear bond strength values after pretreatment of enamel by sandblasting. In a study using casein phosphopeptide-amorphous calcium phosphate (CPP-ACP) and sodium fluoride mouthwash in different application protocols prior to bracket bonding, the evaluation of shear bond strength and ARI scores showed no significant difference between the study groups [36]. It was concluded that the use of CPP-ACP and fluoride can be considered a prophylactic application before bracket placement.

It should be considered that shear bond strength values measured in an in vitro study are normally higher than those measured intraorally during an orthodontic treatment. The difference between these shear bond strength values is on average 57% [41]. A recently published clinical study showed that enamel sealing with a light-cured filled material prior to adhesive bonding of brackets increased the rate of bond failure in the lower dental arch compared to enamel sealing after bracket bonding [27].

The presented results are in partial agreement with recent reports on the influence of SAP P11‑4 on the resin bonding on carious dentine [5, 6]. The authors could show improved bonding between demineralized dentine and composite after pretreatment with SAP P11‑4 if a two-step bonding system was used. The increase in bond strength was presumably due to the increased mineral content of the carious dentine after application of SAP P11‑4. As the present study used enamel with a markedly higher mineral content than demineralized dentin, such improvement was not to be expected and might also not be sought, as it might lead to issues when the brackets are removed from the enamel surface. The positive resistance of the SAP P11‑4 conditioned enamel towards caries has been demonstrated in a recent in situ trial [24]. The in situ trial used a self-assembling peptide matrix (SAPM) gel, which is not suitable to be applied prior to bonding due to additional gel components that cannot be dried in a short time. Yet, various studies could show that SAP P11‑4 leads to an inhibition of demineralization [1, 2, 26].

The present study closed the knowledge gap with regards to the influence of SAP P11‑4 on the bond strength of a metal bracket to the enamel surface. Further research is needed to show the surface characteristics of the enamel after application of SAP P11‑4, e.g., the surfaces hardness or qualitative measurements using a scanning electron microscope [23]. Moreover, comparison of different preventive agents with the SAP P11‑4 prior to bracket bonding should be performed to determine the optimum preventive care for teeth before fixed orthodontic treatment. As a next step clinical investigations are proposed to show the carious inhibitive effect of SAP P11‑4 in the course of orthodontic treatment.

### Conclusion and clinical relevance

The application of the caries protective SAP P11‑4 before bonding did not significantly influence the bond strength of orthodontic brackets regardless of whether they were bonded immediately after application of SAP P11‑4 or after a 24 h mineralization period. Thus, pretreatment of the enamel surface with SAP P11‑4 can be considered before bracket bonding.
