# Correction to: Effect of the caries-protective self-assembling peptide P11-4 on shear bond strength of metal brackets

**DOI:** 10.1007/s00056-021-00307-0

**Published:** 2021-05-21

**Authors:** Thomas Knaup, Heike Korbmacher-Steiner, Anahita Jablonski-Momeni

**Affiliations:** Dental School, Dept. of Orthodontics, Philipps-University Marburg, Georg-Voigt Str. 3, 35039 Marburg, Germany

**Correction to:**

**J Orofac Orthop 2020**

10.1007/s00056-020-00247-1

The article “Effect of the caries-protective self-assembling peptide P11‑4 on shear bond strength of metal brackets”, written by Thomas Knaup, Heike Korbmacher-Steiner, and Anahita Jablonski-Momeni, was originally published electronically on the publisher’s internet portal on 02 September 2020 without open access. With the author(s)’ decision to opt for Open Choice the copyright of the article changed on 22 April 2021 to © The Author(s) 2020 and the article is forthwith distributed under a Creative Commons Attribution 4.0 International License, which permits use, sharing, adaptation, distribution and reproduction in any medium or format, as long as you give appropriate credit to the original author(s) and the source, provide a link to the Creative Commons licence, and indicate if changes were made. The images or other third party material in this article are included in the article’s Creative Commons licence, unless indicated otherwise in a credit line to the material. If material is not included in the article’s Creative Commons licence and your intended use is not permitted by statutory regulation or exceeds the permitted use, you will need to obtain permission directly from the copyright holder. To view a copy of this licence, visit http://creativecommons.org/licenses/by/4.0/.

